# Assessment of Relationship Between Temperature and Selected Technical Parameters of High-Strength, Fine-Grained Ordinary and Polypropylene Fibre-Modified Building Mortars Subjected to Conditions Simulating Fire

**DOI:** 10.3390/ma18235358

**Published:** 2025-11-28

**Authors:** Tomasz Drzymała, Bartosz Zegardło, Krzysztof Przystupa

**Affiliations:** 1 Faculty of Safety Engineering and Civil Protection, Fire University, 52/54 Słowackiego Street, 01-629 Warsaw, Poland; 2 Institute of Computer Science, Faculty of Natural Sciences, University of Siedlce, 3 Maja 54 Street, 08-110 Siedlce, Poland; bartosz.zegardlo@uws.edu.pl; 3 Department of Automation, Lublin University of Technology, Nadbystrzycka 38D, 20-618 Lublin, Poland

**Keywords:** flexural and stretching, cement mortars, cementitious composite, effect of high temperature, mechanical properties, microstructure and mechanical properties, polypropylene fibres, thermal spalling, tensile strength

## Abstract

Cement composites are non-flammable, and their resistance to high temperatures is only apparent. This article presents extensive research on the strength parameters of building mortars exposed to fire-simulating conditions. The analyses included assessment of the mortars’ tensile, compressive and flexural strength, as well as their flexural modulus of elasticity. Microscopic analysis of the samples was performed using a scanning electron microscope (SEM). The results of optimisation studies, particularly tensile strength tests conducted for various types of additives (fibres), showed that the addition of polypropylene fibres had a beneficial effect across the entire temperature range. Based on the research, relationships between temperature and the tested parameters were developed. Polynomial models were applied for their approximation, with the selection justified both by the high consistency with the experimental results and by the nature of the physical changes occurring in the cement mortar during subsequent stages of heating. These models allow an approximate assessment of the condition of mortar after a fire. Based on the conducted microstructural analyses of mortars and their correlation with the strength test results, graphical models were presented to illustrate the phenomena governing the changes in the examined parameters at various fire temperatures. On the basis of conclusions drawn from the analyses, recommendations were formulated regarding the use of polypropylene fibres in selected structural elements that may be exposed to fire, and the limitations of their applicability were indicated.

## 1. Introduction

Emergency situations, including fires regardless of whether they occur in public buildings, residential, industrial or agricultural facilities, pose a number of hazards—not only directly during an incident and rescue operations but also in the post-incident use of these structures. Quite frequently high temperatures lead to significant weakening of the construction, which, especially for structures made of cementitious composites, is difficult to assess without specialised expert evaluations.

Processes that occur in concrete under high-temperature conditions have been a subject of in-depth analyses by numerous researchers [1,2,3,4,5,6,7,8]. The topics addressed in this area of research follow two seemingly related directions. The first one concerns cementitious composites, designed to bear loads at consistently elevated temperatures, as is the case, for example, with the lining of industrial chimneys [9]. The second direction examines the behaviour of materials for which exposure to high temperatures constitutes only an emergency situation, as occurs in the case of fires [10].

In the ready-mix concrete industry, many composites can be found that meet the first criterion. Thanks to the use of special additives in their production, these composites can operate reliably at temperatures reaching up to 1500 °C. A common feature of these materials, however, is the necessity of gradually introducing them to high-temperature conditions. During the successive stages of these processes, transformations occurring within the composites, lasting up to 160 h, ensure their safe operation thereafter [11,12,13]. Although the temperatures affecting composites during emergency fires are lower, their rapid increases make it still difficult to design cementitious composites that would be completely resistant to such impacts.

The effect of high temperatures on the properties of composites, particularly cement composites, is a complex issue given the multitude of phases they contain [14,15]. It has been observed that after a fire, these composites undergo changes in both their external and internal structure, depending on the temperature and duration of the fire [16,17,18,19]. This results in a change in their technical parameters [20]. The mechanical properties of coarse-grained concretes at elevated temperatures depend on changes occurring under the influence of temperature in the structures of the mortar, aggregate and the aggregate−mortar contact zone [21,22,23]. When heating cement composites to temperatures around 400 °C, the most significant effects originate from processes occurring in the cement paste [24,25,26]. At approximately 100 °C, free water evaporates; at around 180 °C, physically bound water is removed; and from ca. 400 °C, due to the decomposition of calcium hydroxide—a component of the crystalline network formed during setting—chemically bound water is released as calcium hydroxide converts into free lime (Ca(OH)_2_ → CaO + H_2_O). Above this temperature, processes occurring in the aggregate become more significant [27]. It is generally accepted that ordinary Portland cement concretes do not significantly lose their strength properties up to about 300 °C. At higher temperatures, their structure gradually deteriorates, leading to reduced strength and increased permanent deformations. In most cases, complete destruction of the concrete occurs only in the temperature range of 500 °C to 600 °C and above [16]. The main cause of strength reduction in heated concrete is the opposing deformation directions of the aggregate and the cement paste, which cause a weakening of the adhesion between them. Such changes are more significant when larger aggregate grains are used in the composite production. Incompatible volumetric changes are accompanied by chemical changes in the structure of the paste and the aggregate. The intensity of their impact on the concrete’s properties depends primarily on the heating rate and the maximum temperature. The cooling method also notably influences the degradation of concrete strength. Elements that have been sprayed with water or immersed in it after heating tend to exhibit a much greater strength loss than those cooling freely in air. In some studies, gradual recovery of strength over time was observed in concrete subjected to heating [27].

Many researchers have conducted studies on the decrease in concrete strength under the influence of high temperatures [28,29,30,31,32,33,34,35,36,37,38]. These studies aimed to observe the phenomena occurring in concrete and to assess the load-bearing capacity of elements. Some of them, such as K. D. Hertz [39], developed formulas allowing for the estimation of strength loss. Numerous series of tests have been carried out on both normal-strength and high-strength concretes. Despite this, it remains difficult to definitively determine the strength reduction depending on the concrete characteristics. Assumptions and comprehensive information regarding testing methods for high-strength concrete (HSC) and normal-strength concrete (NSC) by various researchers are presented in the publication [40].

The behaviour of concrete structures also depends on the flexural modulus of elasticity of concrete, which is considerably influenced by temperature. The effect of elevated temperatures and the type of aggregate on changes in the flexural modulus of elasticity for various concretes has been discussed in [41]. Experimental studies show that concretes with limestone or basalt aggregate are more resistant to high temperatures than those containing aggregates with high silica content. This is largely because, for example, silica sand aggregates melt at temperatures of around 350 °C.

One of the destructive phenomena that lead to the failure of concrete elements under fire conditions is spalling [42,43,44,45,46]. Spalling is described as the flaking off of the surface layers of concrete due to the increase in water pressure within the pores. Boiling water—expanding in volume within the closed capillaries—causes tensile stresses in the capillary walls. When these stresses exceed the tensile strength of the material, they can cause permanent damage such as cracks and fissures. Currently, it is believed that spalling can also arise from other mechanisms, including temperature differences between the surface and the interior of the concrete element, differences in thermal expansion between the aggregate and the cement paste, differences in thermal expansion between the concrete and reinforcement, as well as mineral transformations within the concrete. All of these processes can cause changes both on the surface and inside the volume of the element. The analysis of these phenomena leads to the observation that they become more destructive as the cement composite becomes denser, more compact, and less permeable.

Results from numerous tests conducted on coarse-grained ordinary concretes demonstrate that the addition of polypropylene (PP) fibres can have a positive effect on the behaviour of concrete structures at high temperatures and may contribute to reducing spalling [47,48,49,50]. This has also been confirmed by research reports from the Channel Tunnel Rail Link and the findings of the UPTUN project [37]. The addition of PP fibres influences the mechanical properties of concrete at both normal and elevated temperatures [28]. For ordinary composites, the fibres act as a type of dispersed reinforcement. In elements exposed to high temperatures, the fibres melt and channels formed after their melting enable the escape of water vapour contained in the capillaries.

The latest literature on the degradation of mechanical properties of cement-based composites under high-temperature exposure indicates significant variability in their behaviour, depending on the type of binder, microstructure and the presence of reinforcing fibres [51,52,53,54,55]. In the study by Lima et al. (2025) [51], an analysis was carried out on the effect of temperatures up to 800 °C on lime-cement mortars, employing both destructive and non-destructive methods (UPV). The results showed a drastic degradation in mechanical strength after exceeding 600 °C—compressive strength dropped by approximately 87%, while the ultrasonic pulse velocity (UPV) decreased by 75%. Furthermore, both the static and dynamic modulus of elasticity underwent a greater reduction than the compressive strength itself, indicating a weakening of the stiffness of the material. The authors demonstrated a strong correlation between the values of UPV and mechanical properties, confirming the potential use of non-destructive methods for assessing thermal damage in mortars [51]. Le and colleagues [52] (2025) have investigated alkali-activated slag (AAS) mortars, comparing them with traditional Portland cement (PC) mortars. AAS mortars have demonstrated a 36.9% increase in compressive strength after heating to 200 °C, which was attributed to the further hydration process. However, between 600 and 800 °C, a sharp decrease in both compressive strength and wear resistance was observed, with partial regeneration above 800 °C, when akermanite crystallised. At 1000 °C, thermally cured AAS mortars regained 26.7% of the lost compressive strength, and wear resistance improved by 45%. These results indicate greater thermal stability of AAS compared to that of PC, as confirmed by TGA, XRD and SEM analyses [52]. The most comprehensive analysis of the effect of fibres was presented in the work by Ezziane et al. [53] (2025). The authors examined mortars based on three binding systems (CEM I, CEM I with 8% silica fume addition and CEM III/A based on slag), both plain and fibre-reinforced with steel fibres. It was found that steel fibres increase compressive strength, bending strength and the modulus of elasticity across the entire temperature range. The best results were obtained for the CEM I mortar with silica fume—the improvement in tensile and bending strength was attributed to a more compact microstructure and better fibre-matrix adhesion. At intermediate temperatures (400–500 °C), slag cement mortars (CEM III/A) exhibited increased residual fracture energy, which the authors attribute to the limitation of crack propagation due to the presence of slag. However, at 800 °C, significant oxidation of the fibres and microstructural damage occurred, resulting in a sharp decrease in strength [53]. The study by Han et al. [54] (2025) provided important findings regarding Ultra High-Performance Concrete (UHPC) modified with polyethylene fibres (PEFs), designed to eliminate the phenomenon of explosive spalling. Due to the phase changes of polyethylene fibres (PEFs) (melting and evaporation at around 155 °C), the material imparted a porosity of 5.89% to the composite at 400 °C, which facilitated the dispersion of steam pressure. As a result, the UHPC maintained compressive strengths of 98.3 MPa at 400 °C and 36.0 MPa at 800 °C, completely eliminating the phenomenon of thermal spalling at 1000 °C. The authors developed a quantitative model, indicating that thermal stresses and steam pressure account for 43.6% and 56.4% of the thermal spalling effects, respectively [54]. Similarly, Kong and colleagues (2025) [55] investigated the residual properties of steel-polypropylene fibre-reinforced UHPC subjected to rapid heating. It has been shown that as the content of steel fibres increases, the strength of UHPC rises significantly—particularly below 400 °C, where the fibres effectively limit the development of microcracks and improve structural coherence. Above this temperature, the reinforcing effect diminishes, due to the partial decomposition of polymer fibres and weakening of the interfacial zone. An interesting conclusion was that with faster heating, UHPC exhibited milder degradation during both the increase and decrease in strength, which the authors attributed to the uneven distribution of temperature gradients [55].

By comparing the results of studies [51,52,53,54,55], it can be concluded that mortars and concretes without fibres undergo severe degradation above 600 °C, with a compressive strength loss of 70–90%, while fibre-modified materials (steel, polyethylene and polypropylene) maintain greater structural integrity and a higher residual modulus of elasticity. According to the conducted studies, UHPC with polymer or hybrid fibres achieves the best thermal performance, maintaining strength at levels of several tens of MPa at 800 °C. The use of fibres also limits spalling and improves crack resistance, which is crucial for structures exposed to fire.

These results confirm that the selection of appropriate types of fibres and binder compositions (e.g., with the addition of slag or silica fume) can significantly enhance the fire resistance of concrete and mortars, improving their performance under extreme temperatures. Ongoing research into the use of a wide range of admixtures, additives and diverse cement composite structures remains relevant and justified. In light of these findings, it becomes particularly important to conduct studies on the effectiveness of polypropylene fibres in typical building mortars, which still constitute the primary material for many structural elements, as well as developing relationships that enable the assessment of their condition after a fire.

This study addresses the issue of fire resistance in high-strength, fine-grained construction mortars. The composition of cement mortars is similar to that of concrete. Mortars also consist of cement paste and aggregate, but with finer grain sizes. Therefore, the temperature distribution within the mortar is more uniform. Similarly, the use of construction mortars—which are employed to create load-bearing thin-walled elements, masonry bonds and plasters—allows a more even temperature load under natural service conditions. From the perspective of potential damage caused by differential thermal expansion of the individual phases or layers of the composite, this factor should be minimised. The main cause of mortar deterioration under high-temperature exposure, as identified by the authors, may be their excessive impermeability and the common use of silica sands in their production. The primary hypothesis of the proposed study assumed that modifying high-strength traditional mortars with polypropylene fibres would improve their strength parameters and that dispersed reinforcing fibres would beneficially affect the tensile, compressive and flexural strength of the mortar, as well as its flexural modulus of elasticity, even under fire conditions. During the analyses, the mentioned technical parameters of the mortars were evaluated as they were successively exposed to temperatures of 100 °C, 200 °C, 300 °C, 400 °C, 500 °C and 600 °C. Ordinary mortars without fibre addition were used as the reference material.

## 2. Materials and Methods

### 2.1. Materials Used in the Study

For the preparation of the mortar, Lafarge CEM I 42.5 R (Holcim Group, Bielawy, Poland) cement was used, which, according to the manufacturer’s declaration, meets the requirements of the standard [56] PN-EN 197-1:2002 “Cement. Part 1: Composition, specifications and conformity criteria for common cements”. This cement is characterised by stable physicochemical parameters, appropriate setting time, high early and final strength parameters, low alkali content and high resistance to aggressive chemical agents, making it commonly used in the production of ready-mix concrete. Detailed values of the cement’s physicochemical parameters are summarised in Table 1.

The mortar also utilised Wisła sand with a grain size of 0/2 mm and Silimic (Silimic, Łaziska Górne, Poland) silica fume. Silica fume is a by-product obtained during the production of metallic silicon and ferrosilicon alloys in electric arc furnaces. It consists of fine dust particles, the diameter of which is estimated to be on average one hundred times smaller than the average grain size of cement. According to the manufacturer’s data, replacing 15% of the cement with silica fume increases the impermeability of concrete by several dozen times, which is difficult to achieve by other methods. Additionally, compressive strength is increased by 20%, and water absorption is reduced threefold. In terms of modifying composites with silica fume that may be exposed to high temperatures, this additive is viewed as unfavourable. The increased impermeability of the cementitious composite hinders the free evaporation of water contained in the capillaries. Water expanding in volume can cause tensile stresses in the capillary walls, which, once the tensile strength is exceeded, leads to the destruction of the composite. To utilise the primary benefits of silica fume, it was decided to use it nonetheless. The basic properties of silica fume, taken from its technical data sheet, are presented in Table 2.

Tap water compliant with the requirements of [61] PN-EN 1008:2004 “Mixing water for concrete—Specification for sampling, testing and assessment of mixing water” was used for the mortar. As an admixture, Chrysofluid Optima (Chryso Poland Ltd., Błonie, Poland) 185 was used, meeting the requirements of the standard [62] PN-EN 934-2 “Admixtures for concrete, mortar and grout—Part 2: Concrete admixtures—Definitions, requirements, conformity, marking and labelling”. Chrysofluid is a high-performance plasticising admixture mainly used for the production of high-strength ready-mix concrete, self-compacting, and easily compactable mixes. According to the manufacturer, it plasticises and homogenises the concrete mix, maintains consistency over a long period and improves the early strength of concrete. The basic properties of the admixture, as taken from its technical data sheet, are presented in Table 3.

**Figure 1:**
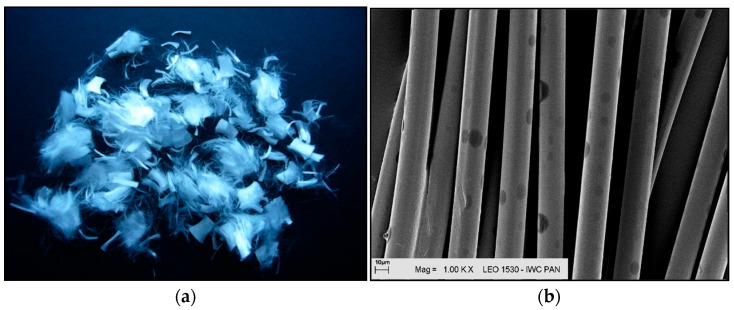
Polypropylene fibres “I” (Ignis): (**a**) photograph without magnification; (**b**) SEM photograph.

**Figure 2:**
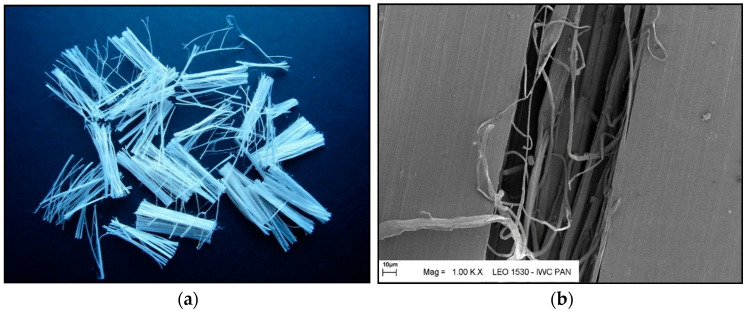
Polypropylene fibres “F” (Fibrofor Fibre High Grade 190): (**a**) photo without magnification; (**b**) SEM photo.

For the tests, two types of polypropylene fibres, also used as additives in concrete, were employed as additives and modifiers, designated for research purposes as I and F (Figure 1 and Figure 2)—polypropylene fibres I, with the trade name Ignis^®^ (PP Nordica, Zgorzelec, Poland) and fibres F, with the trade name Fortatech^®^ Fibre High Grade 190 (currently known as Fibrofor Fibre High Grade 190 from Contec Fiber AG, Domat, Switzerland). The fibres mainly differed in the length and thickness of individual fibres. Fibre I is a monofilament fibre, meaning it is made from a single filament. Fibre F is a multifilament fibre, meaning it is formed by twisting several individual filaments together. Polypropylene fibres I and F serve as dispersed micro-reinforcement in the form of fibres with lengths of approximately 12–19 mm and diameters of 18–40 μm.

The purpose of adding fibres was to strengthen the structure of unheated composites, in which they were intended to act as dispersed reinforcement. In composites exposed to high temperatures, they were meant to compensate for the mortar’s impermeability. The channels formed after their melting were expected to allow the evaporation of residual capillary water. Table 4 presents the basic properties of the fibres based on the manufacturer’s data.

As a model research material, a cement mortar with a low water-to-binder ratio (w/b = 0.23) was adopted, densified with the addition of the aforementioned silica fume. Its composition was provided by the contractor, who uses it for the production of construction products. The research presented in the article was aimed at assessing the possibility of improving the strength parameters of cement composites subjected to high temperatures by introducing polypropylene (PP) fibres into the mix. Thus, the composition of the base mortar for determining tensile strength was modified by adding polypropylene fibres “I” and “F” in the literature-suggested amount of 1.8 kg/m^3^. The amount of the additive was also dictated by the authors’ own experience based on unpublished work. In these studies, optimisation analyses were conducted regarding the use of fibres in quantities of 1.8 kg/m^3^, 3.0 kg/m^3^ and 3.6 kg/m^3^. The conclusions drawn from these pilot studies indicated that in composites with a higher fibre content, after their thermal destruction, the voids occupy too much of the composite volume, which negatively affects the technical parameters at higher temperatures.

As regards the remaining strength tests, the optimal composition determined for fibre F was used. The established optimal dosage of PP fibres was confirmed during the first stage of laboratory tests discussed later in the article, as well as in the authors’ previous research on concrete composites. This amount of additive was also considered optimal for the mortar.

### 2.2. Sample Preparation

The samples for testing were prepared at the Concrete Technology Laboratory in the Department of Building Materials Engineering at the Faculty of Civil Engineering, Warsaw University of Technology. Prior to testing, cement mortar mixes with and without the addition of polypropylene fibres were designed. Within each test series, mortar samples were produced based on the same recipe, which is presented in Table 5.

All mortar mixes were prepared in a laboratory mixer. First, the dry ingredients (sand, cement) were measured and mixed for approximately 60 s. Next, the polypropylene fibres were added and mixed for 30 s (this short mixing time was due to fibres clumping and settling on the mixer blades during longer mixing). Then, water and an admixture in an amount of 2% of the cement mass were added. All ingredients were mixed further for about 3 min. The samples were formed and compacted in two layers on a vibrating table for approximately 15 s. The formed samples were placed on a flat laboratory floor and tightly covered with foil to prevent excessive evaporation of water from their surface. After 24 h, the samples were removed from the moulds and placed in water for the next 27 days. After this period, the samples were stored in a climate chamber for 60 days at a temperature of 20 °C and 99% humidity. Subsequently, the samples were placed in a dryer and dried at 70 °C to constant mass over 21 days, with water loss monitored throughout this time. The drying of the tested mortar samples to a constant mass before the heating process was due to the fact that for wet samples without the addition of PP fibres, during heating to 600 °C, numerous spalling explosions were observed, which could have significantly damaged the testing equipment. Each sample was thoroughly documented, including the date of forming, and was assigned a unique number (the sample number corresponded to a specific test recorded on the control sheet, thereby avoiding possible errors). All samples were formed and stored identically until the time of testing. In order for the test results to be reliable, 11 samples were prepared for each type of specimen in every test.

### 2.3. Methods

Tensile strength tests were carried out on samples shaped as “figure-eights”. Flexural and compressive strengths of the mortar were determined on prismatic samples with nominal dimensions of 40 × 40 × 160 mm. Splitting tensile strength tests were performed on cubic samples with nominal dimensions of 100 × 100 × 100 mm.

**Figure 3:**
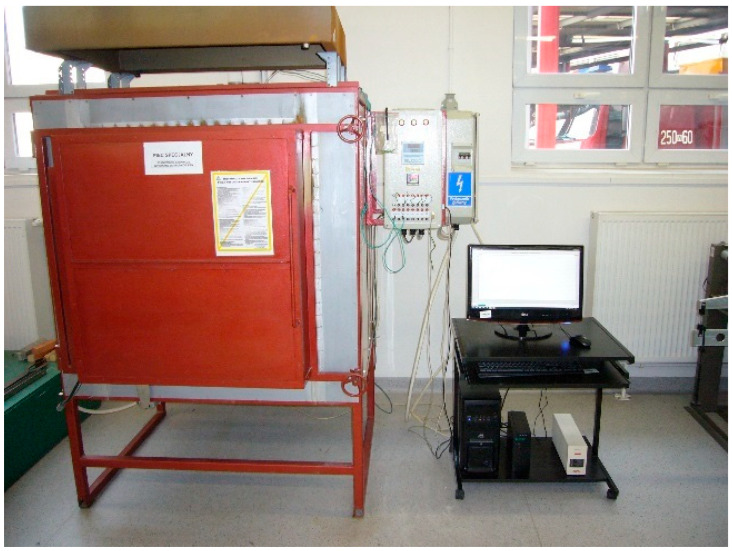
Sample annealing station.

The fire exposure simulation was conducted at the Applied Mechanics Laboratory at the Fire Academy. The setup for heating the samples consisted of an electric medium-temperature chamber furnace, model PK 1100/5, along with a PC (Figure 3). Furnace control and temperature recording during sample heating were carried out using specialised software.

After the designated conditioning period, the samples of each composite were divided into groups subjected to the heat treatment process. The test temperatures ranged from 20 °C to 600 °C. The experimental work programme set the maximum temperature as a level at which significant changes can be caused by polypropylene fibres. Up to this temperature, the progress of destruction and disappearance of the PP fibres can be observed. Due to the fact that at higher temperatures, the fibres completely disappear and their influence on the composite is no longer directly noticeable, the scope of the research was limited to 600 °C. Ultimately, the samples were heated in a furnace at six test temperatures (100 °C, 200 °C, 300 °C, 400 °C, 500 °C and 600 °C). During the tests, efforts were made to ensure that the temperature profile over time closely matched the thermal conditions of a standard fire.

To measure temperature during the heat treatment process, three thermocouples were used: a control thermocouple (TR) measuring the furnace temperature and two thermocouples measuring the temperature on the sample (T1, T2). Thermocouple T1 was attached to the surface of the sample, while thermocouple T2 was placed inside a drilled channel—its end positioned halfway through the thickness of the sample. At each temperature, a batch of samples intended for testing was heated in the furnace, along with an additional sample on which the temperature was measured using thermocouples T1 and T2. The procedure was carried out in accordance with the guidelines of standard [64] PN-EN 1991-1-2 Eurocode 1: Actions on structures, Part 1-2: Actions on structures during fire (Polish Committee for Standardization, Warsaw, 1991). The samples were heated until the thermocouples reached the target temperature during the time of ca. 120 min. After the temperatures on the thermocouples stabilised, the set temperature was maintained for an additional 30 min. Each time after heating, the furnace was turned off and cooled down to a safe temperature (approximately 100 °C); then, the furnace was opened and the samples were allowed to cool for about 24 h until they reached room temperature (20 °C).

**Figure 4:**
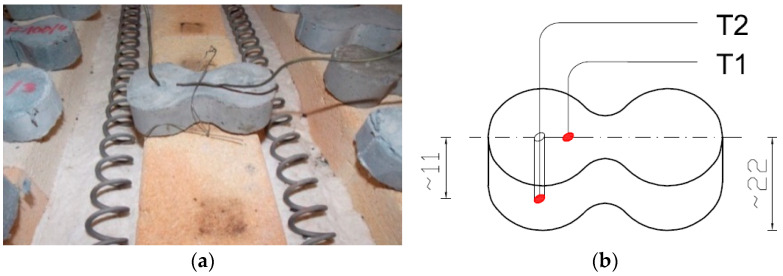
Arrangement of measurement thermocouples in the “figure-eight” specimen: (**a**) view of the specimen with attached thermocouples [63]; (**b**) schematic layout of thermocouples within the specimen.

The first stage of the research—optimisation tests—involved tensile strength testing conducted on “figure-eight”-shaped samples. Similar to what was described above, for each batch, apart from the sample equipped with measuring thermocouples, sets of samples containing two types of fibres at a dosage of 1.8 kg/m^3^ were heated. These prepared sample batches were heated at six different temperatures (100 °C, 200 °C, 300 °C, 400 °C, 500 °C and 600 °C). The reference temperature was set at 20 °C (see Figure 4a,b).

**Figure 5:**
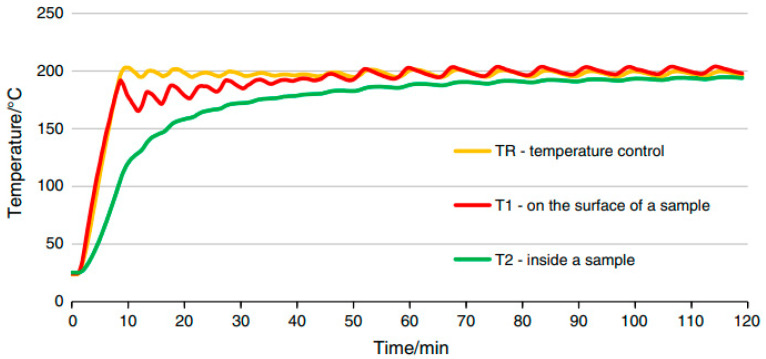
Example of heat treatment of the concrete sample at 200 °C [63].

Figure 5 shows an example curve illustrating the actual temperature distribution on an octagonal sample at the locations of the measuring thermocouples [64,65,66].

**Figure 6:**
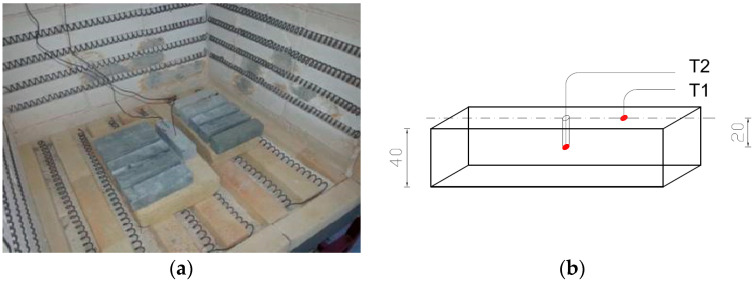
Arrangement of measuring thermocouples in a sample with nominal dimensions of 40 × 40 × 160 mm: (**a**) view of the sample with thermocouples attached; (**b**) diagram of the arrangement of thermocouples in the sample.

**Figure 7:**
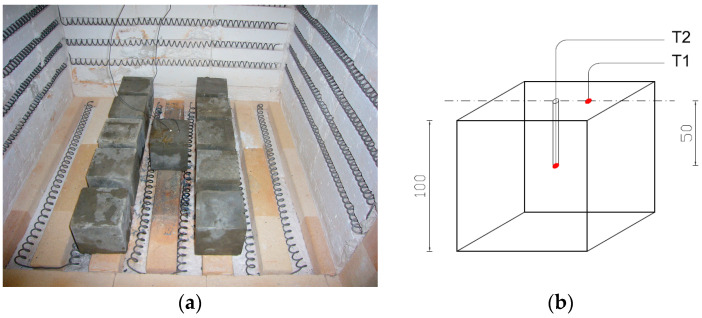
Arrangement of measuring thermocouples in a sample with nominal dimensions of 100 × 100 × 100: (**a**) view of the sample with thermocouples attached; (**b**) diagram of the arrangement of thermocouples in the sample.

Figure 6 and Figure 7 demonstrate a view of the furnace chamber with the arrangement of sample batches intended for testing flexural strength, flexural modulus of elasticity and splitting tensile strength.

Strength tests were carried out at the Department of Building Materials Engineering at the Warsaw University of Technology.

**Figure 8:**
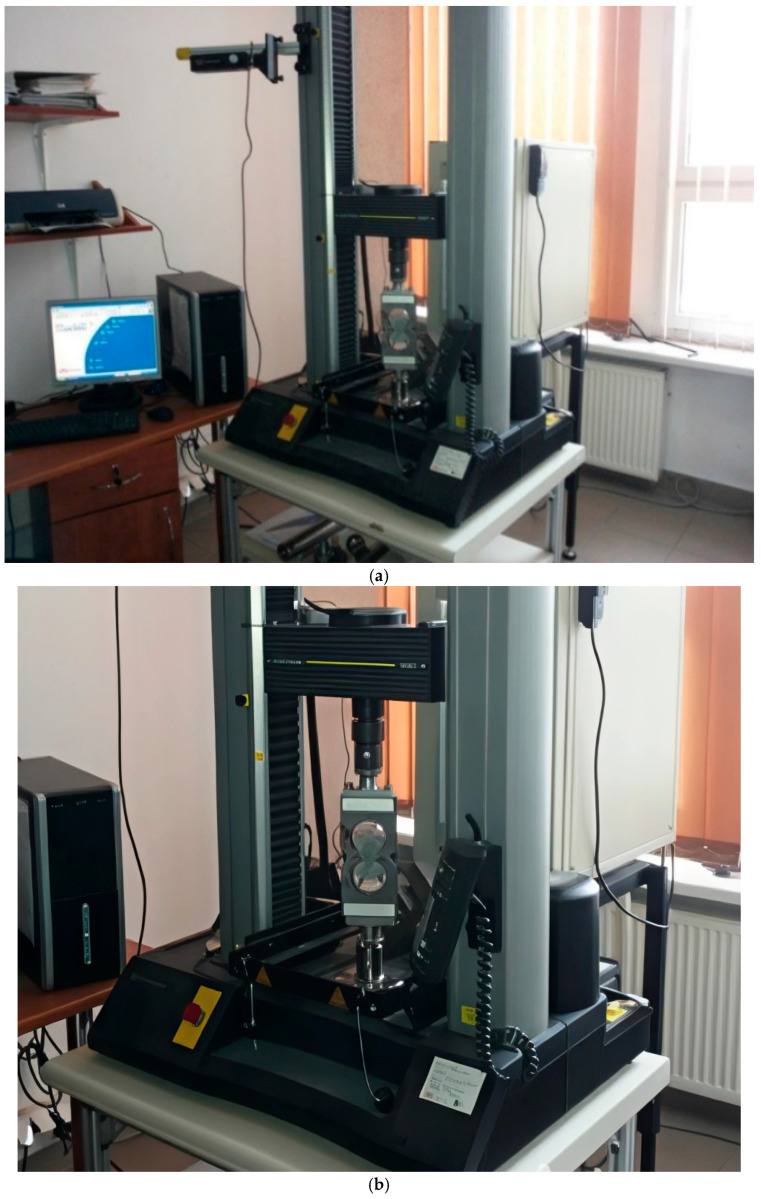
Tensile strength test rig: (**a**) measurement stand; (**b**) close-up of the jaws of the measuring machine.

Optimisation tests—tensile strength tests were conducted on “figure-eight”-shaped specimens. The tensile strength determination was carried out according to standard [67] PN-85 B-04500: Construction mortars—Testing of physical and strength characteristics. The INSTRON 5567 testing machine with a measurement range of 0–30 kN was equipped with curved grips for holding the figure-eight specimens (Figure 8). The figure-eight specimens for testing were produced using special removable steel moulds that met the standard requirements.

The flexural and compressive strength of the mortar were established on prismatic specimens with nominal dimensions of 40 × 40 × 160 mm according to the procedure described in the standard [68] PN-EN 1015-11:2001: Methods of testing mortar for masonry—Part 11: Determination of flexural and compressive strength of hardened mortar.

The determination of splitting tensile strength of cubic samples with nominal dimensions of 100 × 100 × 100 mm was performed according to the standard [69] PN-EN 12390-6:2011 Testing of concrete—Part 6: Tensile splitting strength of test specimens. The determination of splitting tensile strength of cement mortars with and without the addition of polypropylene fibres was conducted using a suitably adapted CONTROLS MCC8 strength testing machine.

**Figure 9:**
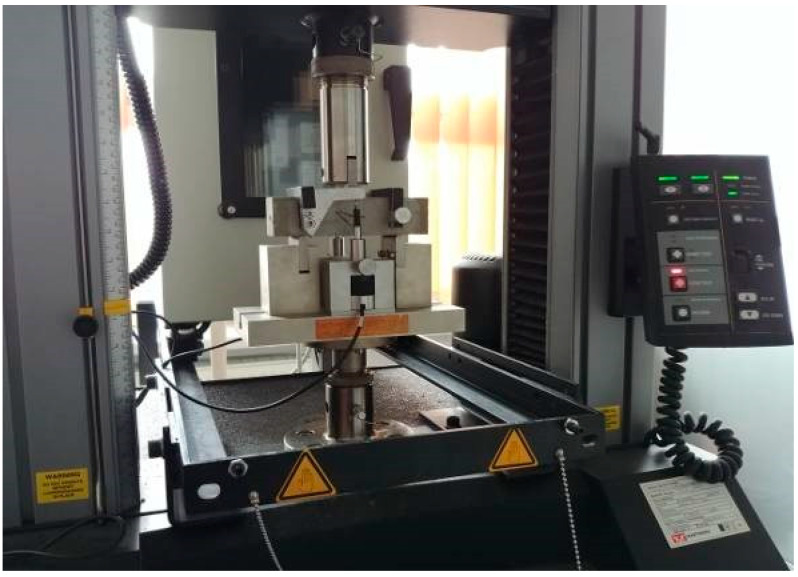
Station for determining the elastic flexural modulus using the 3-point flexural method on 40 × 40 × 160 mm beams.

The flexural modulus of elasticity was determined using a specially adapted INSTRON 5567 testing machine with a measurement range of 0—30 kN. The flexural modulus of elasticity in flexural was established using the three-point flexural method (Figure 9).

**Figure 10:**
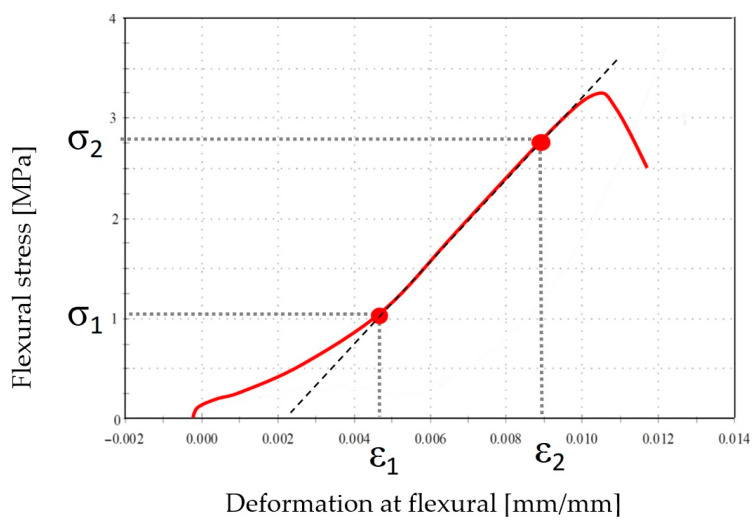
Stress−strain relationship obtained during the flexural test (red line—actual measurement result, dashed line—stress-strain curve in the elastic range, where Hooke’s law applies).

The measurement is typically performed using a computer-controlled testing machine equipped with a strain gauge system for displacement measurements. The flexural modulus of elasticity in flexural corresponds to the tangent of the slope of the stress−strain curve in the elastic region, where Hooke’s law applies (Figure 10).

This region is preliminarily determined by excluding the initial and final parts of the curve due to the settling of the specimen in the testing device at the beginning of the test and the nonlinear behaviour of the curve near the point of specimen failure. The specimens prepared for the flexural strength tests were shaped as beams measuring 40 × 40 × 160 mm.

## 3. Results

**Figure 11:**
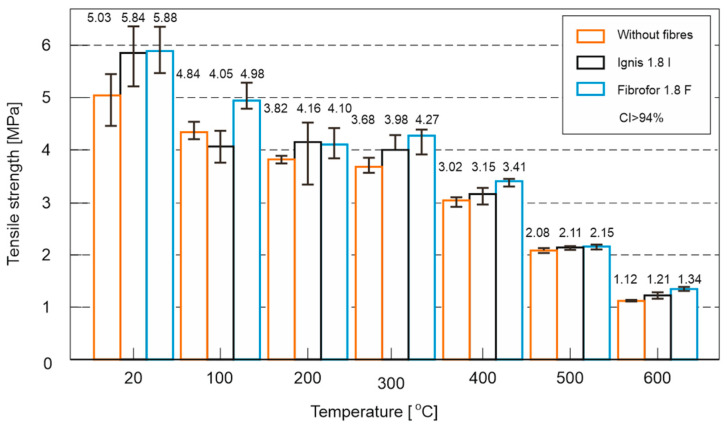
Results of the optimisation study—tensile strength tests performed on different types of fibres, which were conducted on samples in the form of figure eights.

The results of the optimisation study—tensile strength tests performed on different types of fibres, which were conducted on “eight” samples—are presented in Figure 11.

An analysis of the test results has demonstrated a clear influence of both temperature and fibre quality on the mechanical properties of the material. Regardless of the composition, a systematic decrease in strength values was observed with increasing temperature; however, the use of fibres in most cases allowed partial mitigation of concrete degradation.

At the reference temperature (20 °C), the addition of both types of fibres improved strength by approximately 16% compared to control samples without fibres, indicating a positive effect of dispersed reinforcement in the initial state. A different situation occurred at 100 °C: while samples with Ignis fibres exhibited a strength decrease of about 7% compared to the control series, the use of Fibrofor fibres caused a significant increase—up to 15% relative to the concrete without additives.

The result of this test, even at this relatively low elevated temperature, has consequently demonstrated different behaviours of the two fibre types. Considering that at this temperature the fibres are not yet fully thermally degraded, the described situation was likely influenced by their structure and the benefits resulting from the multifilament construction of Fibrofor fibres. The F fibre is a multifilament fibre, meaning it is formed by twisting several single filaments together, which probably affected both the fibre’s strength and its better adhesion to the cement matrix.

In the temperature range of 200–400 °C, a clearly beneficial effect of the fibres was observed. Both Ignis and Fibrofor increased strength compared to the reference samples, with Fibrofor being more effective, especially at 300 °C (+16%) and 400 °C (+13%). In contrast, the corresponding increases for Ignis fibres were lower: 300 °C (+8.5%) and 400 °C (+4.5%).

At higher temperatures, i.e., 500 °C and 600 °C, the differences between the series significantly decreased due to the ongoing degradation of both the cement matrix and the fibres themselves. Nevertheless, even in this range, the series with fibres showed an advantage over the control samples, particularly at 600 °C, where the use of Fibrofor fibres helped maintain strength approximately 20% higher compared to fibre-free concrete. Ignis fibres also had a beneficial effect at these temperatures, although their impact was less pronounced (+8%).

In summary, the results clearly indicated the positive role of dispersed reinforcement in increasing the resistance of concrete to elevated temperatures. Fibrofor fibres were found to have greater effectiveness across the entire tested temperature range, providing both higher initial strength and more stable behaviour during heating. Ignis fibres also positively influenced the mechanical properties; however, their effect was less stable, particularly around 100 °C, where a decrease in strength was observed compared to the control sample.

Taking the above into account, further research was conducted on samples considered optimal, containing Fibrofor fibres at a dosage of 1.8 kg/m^3^, hereinafter referred to as 1.8F. The results were compared to reference samples without fibres, designated as 0F.

**Figure 12:**
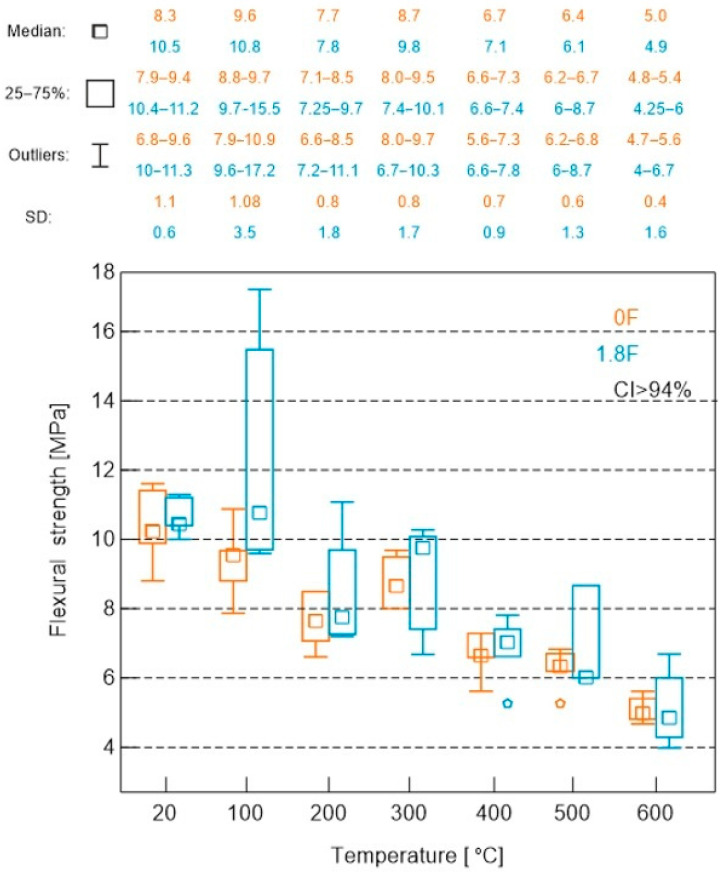
Results of flexural strength tests on 40 × 40 × 160 mm cuboid specimens.

The results of flexural strength tests on 40 × 40 × 160 mm cuboid specimens are presented in Figure 12.

An analysis of the average flexural strength of mortars exposed to high temperatures has demonstrated an increase in strength for both the tested and comparative mortars in the range up to 100 °C, a decrease at 200 °C, an unusual increase at 300 °C and a decrease at higher temperatures. Analyses of the tensile strength test results also demonstrated a clear positive effect of the addition of polypropylene fibres on this parameter. The value of this parameter tested for non-heated composites was 20% higher for the composite with fibres than for the composite without fibres. In the case of values tested at successively increasing temperatures, only at 300 °C was a slightly lower tensile strength value obtained, 0.1 MPa less than in the case of the fibre-free composite. In the other cases, the values for the PP composite were higher and amounted to 25% at 100 °C, 10% at 200 °C, 1% at 400 °C and 1% at 600 °C.

The analysis of the average flexural strength of mortars subjected to high temperatures has shown, for both the tested mortar and the reference mortar, an increase in strength up to 100 °C, a decrease up to 200 °C, an atypical increase at 300 °C and a decrease at higher temperatures. The analysis of the tensile strength test results also demonstrated a clear positive effect of the addition of polypropylene fibres on this parameter. The value of this parameter measured for the unheated composites was 20% higher for the composite with fibres compared to the composite without fibres. For the values measured at increasing temperatures, only at 300 °C was a slightly lower tensile strength recorded, being 0.1 MPa less than that of the composite without fibres. In all other cases, the values for the composite with PP were higher, amounting to 25% at 100 °C, 10% at 200 °C, 1% at 400 °C and 1% at 600 °C.

Based on the presented results of strength values as a function of temperature, a mathematical model was developed to enable an approximate assessment of parameters after exposure to high temperature. Among the tested relationships (2nd- and 3rd-degree polynomials and an exponential model), the best fit was obtained for the 3rd-degree polynomial, which had a coefficient of determination of *R*^2^ = 0.843.

The relationship is described by the equation:

(1) $$R\left(T\right)\approx 2.42\cdot 10^{-8}T^{3}-3.58\cdot 10^{-5}T^{2}+7.23\cdot 10^{-3}T+8.43$$

where

*R(T)*—the strength [MPa];

*T*—the temperature [°C].

**Figure 13:**
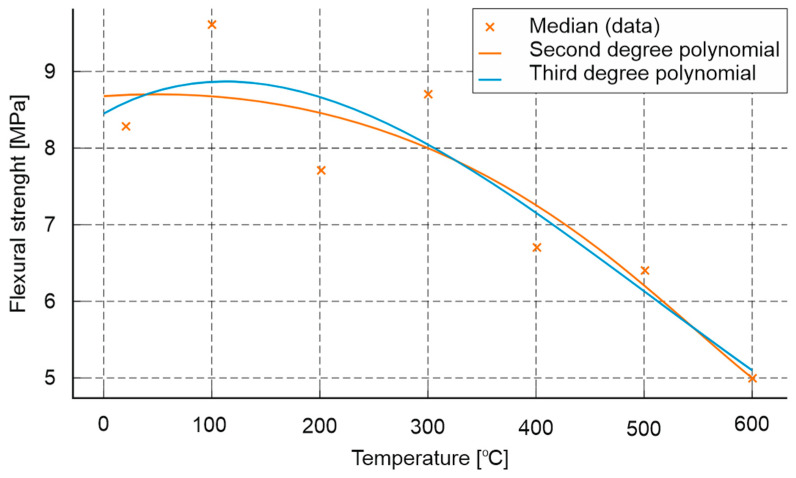
Mathematical model enabling approximate assessment of the impact of high temperature on the flexural strength of cement mortars.

The resulting model (Figure 13) accurately reflects the initial slight increase in strength up to 100 °C, followed by a systematic decrease with increasing temperature, confirming its usefulness for the approximate assessment of material degradation under fire conditions.

It should be noted that the use of this type of approximation is empirical, and its aim is not to directly replicate the physical phenomena occurring in the material, but rather to provide an approximate description of the trend of changes based on the obtained measurement results. In materials research, particularly regarding changes in mechanical properties as a function of temperature, a nonlinear behaviour of the curves is often observed. For this reason, polynomial functions are commonly used for local interpolation or extrapolation of data, allowing the determination of intermediate values, e.g., within a temperature range where no measurement was taken. In this study, polynomial models were chosen as a compromise between simplicity of notation and sufficient accuracy in replicating the experimental measurement points (the coefficient of determination, *R^2^*, for the third-degree model exceeds 0.98). The analysed functions accurately reflect the downward trend in strength with increasing temperature, as well as local deviations in lower temperature ranges, which confirms their usefulness for approximate assessment of parameters after failure or under operating conditions. It should also be emphasised that the applied model is descriptive (empirical) rather than predictive in a physical sense—it does not directly reflect the mechanisms of material structure degradation but allows for their quantitative approximation within the investigated temperature range.

**Figure 14:**
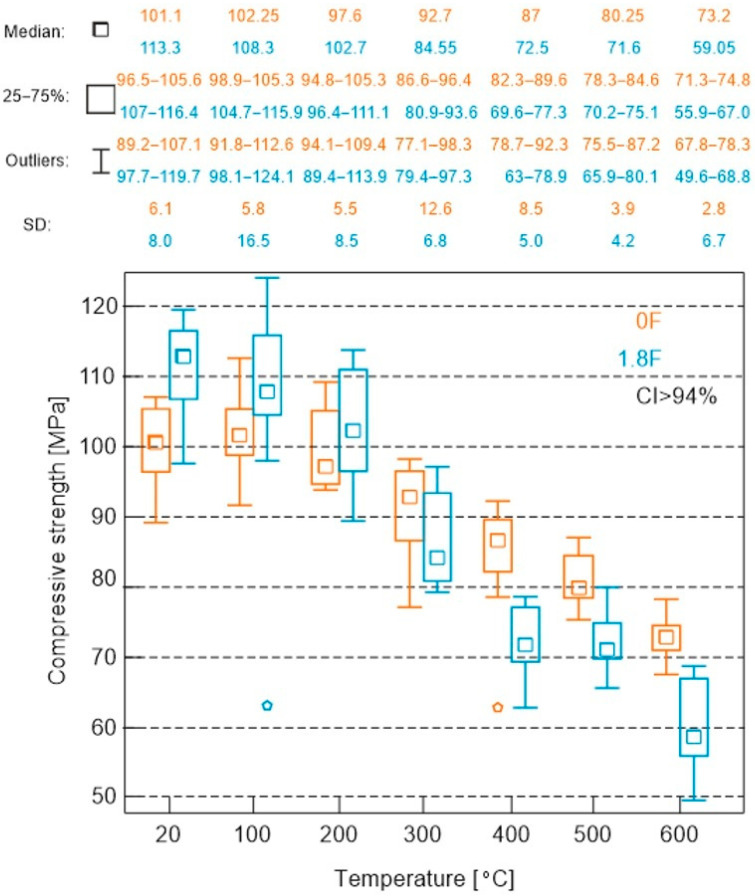
Compressive strength test results.

The results of the compressive strength test are presented in Figure 14.

In the analysis of compressive strength test results for both types of mortars, an almost uniform linear decrease in compressive strength was observed with increasing temperature, with a slight increase in strength around 100 °C. The initial value for the composite without polypropylene (PP) was 99.9 MPa and it decreased almost linearly to 73.1 MPa at 600 °C. The only exception to this trend was a slight increase in strength at 100 °C, reaching 105.8 MPa. For the composite with PP, the decrease ranged from a value of 110.6 MPa at room temperature to 60.3 MPa at 600 °C. The influence of the additive was not unequivocal. Up to a temperature of 200 °C, the effect of PP was beneficial, and at the analysed temperatures, the compressive strength of the composite with PP was correspondingly higher by 15% at 20 °C, 10% at 100 °C and 7% at 200 °C. Beyond this point, at higher temperatures, a decrease in compressive strength was observed. For the composite with PP, the values were lower by 10% at 400 °C, 10% at 500 °C and 10% at 600 °C.

**Figure 15:**
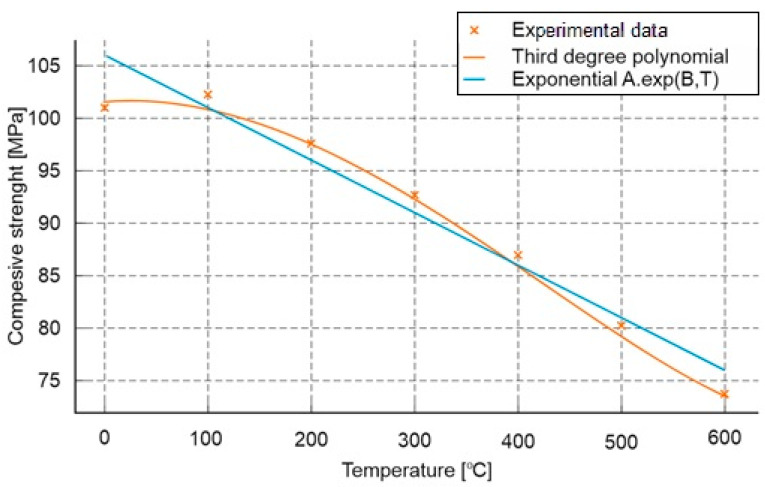
Mathematical model enabling approximate assessment of the impact of high temperature on the compressive strength of cement mortars.

Based on the analysis of the test results, an estimated formula for determining the post-fire load-bearing capacity of mortars subjected to fire conditions was also developed (Figure 15).

Two mathematical models were used to approximate the results:

− Third-degree polynomial:
  (2) $$Rc\left(T\right)\approx 1.29\cdot 10^{-7}T^{3}-1.80\cdot 10^{-4}T^{2}+1.50\cdot 10^{-2}T+101.34$$
− Exponential model:
  (3) $$Rc\left(T\right)\approx 107.13\cdot e^{-5.76\cdot 10^{-4T}}$$

where

*Rc(T)* denotes the compressive strength [MPa] as a function of temperature *T*[°C].

The fit of both models was evaluated using the coefficient of determination *R*^2^ and the root mean square error *(RMSE).* The polynomial model achieved a very high agreement with the experimental data (*R*^2^ = 0.997, *RMSE* = 0.54 MPa), clearly outperforming the exponential model (*R*^2^ = 0.936, *RMSE* = 2.56 MPa). This indicates that within the tested temperature range, the relationship is nonlinear, and taking into account higher-order variations better reflects the actual degradation curve of strength.

The curve shows that the maximum compressive strength occurs at a temperature of around 100 °C (102.25 MPa), which can again be linked, as in the case of the flexural strength test, to the processes of free water removal and temporary densification of the structure. Above this temperature, a systematic decrease in load-bearing capacity is observed—down to about 73 MPa at 600 °C, corresponding to a loss of approximately 28% compared to the initial value.

**Figure 16:**
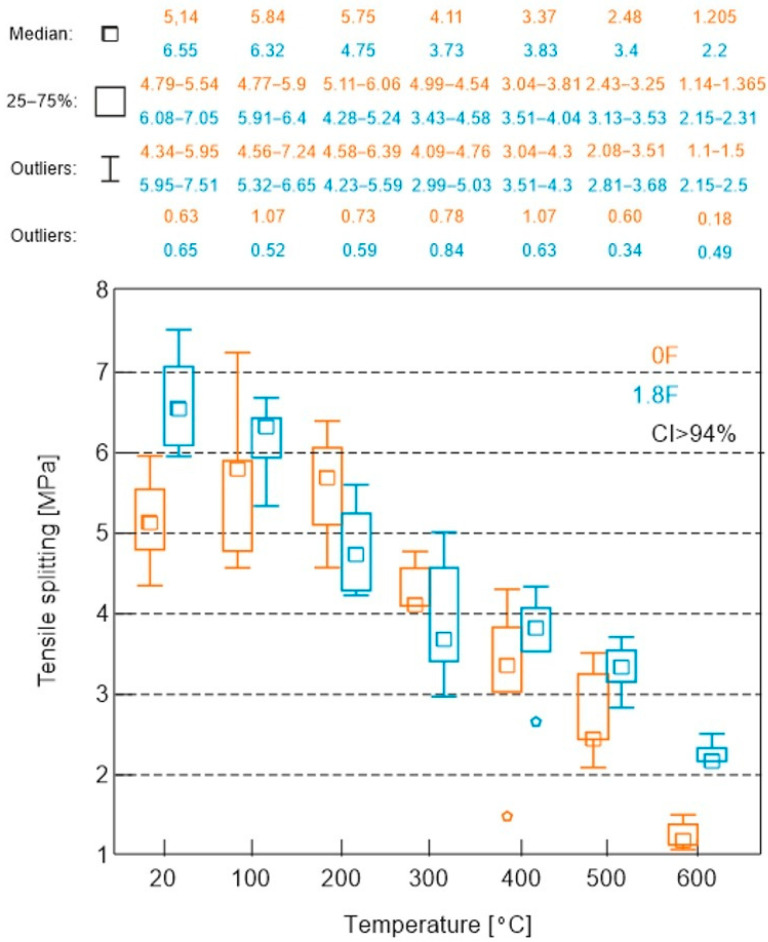
Results of splitting tensile tests on cubic specimens with nominal dimensions of 100 × 100 × 100 mm.

The results of the splitting tensile test on cubic samples with nominal dimensions of 100 × 100 × 100 mm are presented in Figure 16.

In an analysis of the splitting tensile strength results for the mortar with polypropylene fibres, an almost uniform linear decrease in strength was observed with increasing temperature. The initial value for the composite with polypropylene was 6.63 MPa, and it decreased nearly linearly to 2.08 MPa at 600 °C. For the composite without polypropylene, an increase in strength was noted at 100 °C, but at higher temperatures, the strength decreased to a very low value of 1.25 MPa.

Regarding this tested parameter, similar to the tensile strength test, the addition of polypropylene was generally beneficial. Most of the measured values across the entire temperature range were higher for the mortar with polypropylene. The only exception was for the mortars tested at 200 °C and 300 °C, where higher values—by approximately 3% and 5%, respectively—were recorded for the mortars without polypropylene.

**Figure 17:**
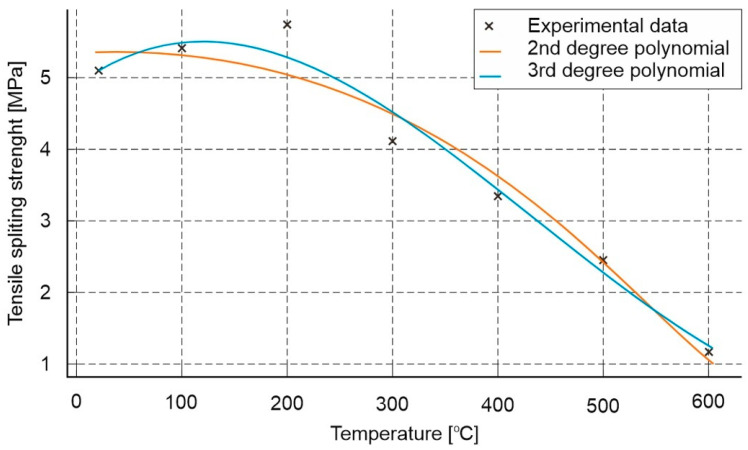
Mathematical model enabling approximate assessment of the impact of high temperature on the splitting tensile strength of cement mortars.

Based on the analysis of the test results, similar to the earlier results, an approximate model was developed that allows the estimation of changes in tensile strength during the splitting of cement mortar subjected to high temperatures (Figure 17).

Two mathematical models were used to fit the experimental data:

Second-degree polynomial:

(4) $$R_{t}\left(T\right)\approx -1.42\cdot 10^{-5}T^{2}+1.36\cdot 10^{-3}T+5.35$$

Third-degree polynomial:

(5) $$R_{t}\left(T\right)\approx -4.36\cdot 10^{-8}T^{3}-5.48\cdot 10^{-5}T^{2}+1.11\cdot 10^{-2}T+4.93$$

where

*R_t_(T)* denotes the tensile strength at splitting [MPa] as a function of temperature *T* [°C].

The fitting of both models was assessed using the coefficient of determination, R^2^. The third-degree model exhibits a better fit to the experimental data (*R^2^* = 0.974) as compared to the second-degree model (*R*^2^ = 0.955).

The obtained relationships indicate a nonlinear nature of strength degradation as a function of temperature. In the range up to approximately 200 °C, a slight increase in strength is observed, which can be associated with the evaporation of free water and partial densification of the structure. Above this temperature, a systematic decrease in strength occurs—down to approximately 1.2 MPa at 600 °C, corresponding to a loss of about 75% of the initial load-bearing capacity.

**Figure 18:**
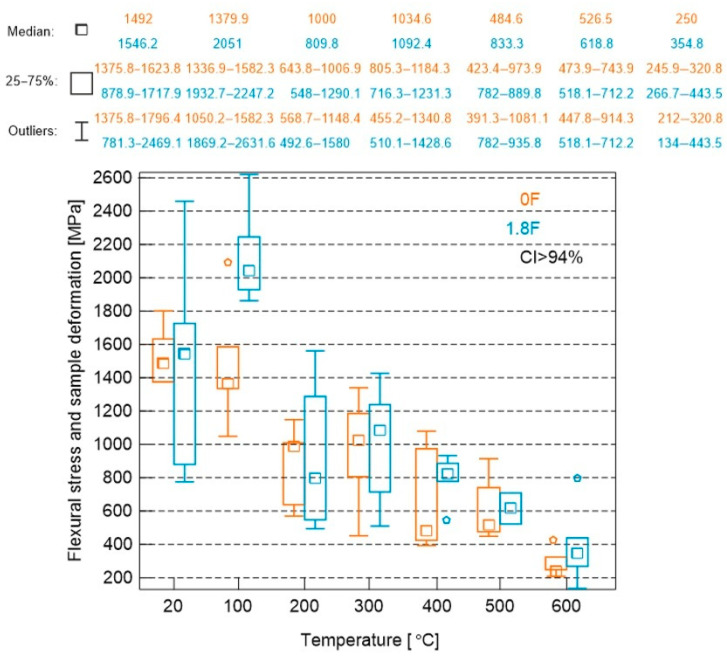
Results of tests on the relationship between flexural stress and sample deformation.

The results of the tests on the relationship between flexural stress and sample deformation are shown in Figure 18.

**Figure 19:**
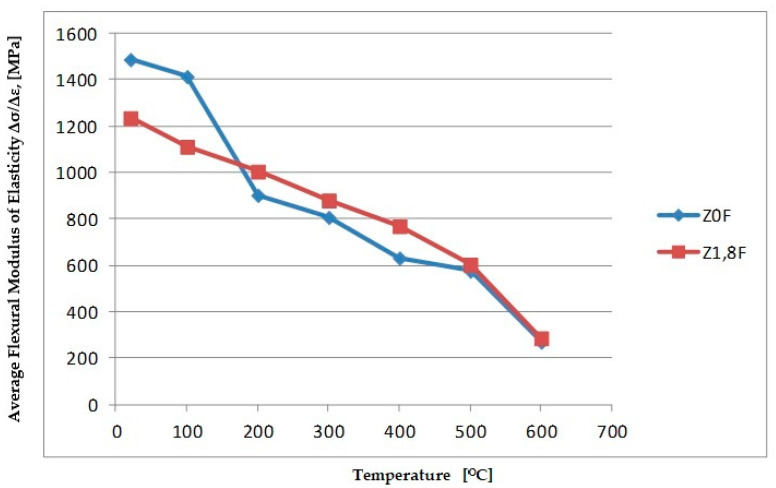
Flexural modulus of elasticity in flexion.

The results of the study of the relative change in flexural elasticity modules are given in Table 6 and Figure 19.

The test results indicate that, with increasing heating temperature, the flexural modulus of elasticity of the tested cement mortars decreases. This trend was observed for both the samples containing polypropylene fibres and those without fibres. In the case of flexural modulus testing, it was noted that the fibre-free samples initially exhibited a higher flexural modulus of elasticity. This was observed at 20 °C and 100 °C. Above these temperatures, starting from 200 °C, a relatively significant reduction in the flexural modulus was recorded (around 40%), whereas the fibre-reinforced samples showed a smaller decrease (only about 19%). In the temperature range of 200 °C to 600 °C, the flexural modulus of elasticity of the fibre-free samples was lower than that of the mortars that contained fibres. At 600 °C, both types of mortars showed a similarly high reduction in flexural modulus values—approximately 82% for mortars without fibres and around 77% for those containing fibres.

**Figure 20:**
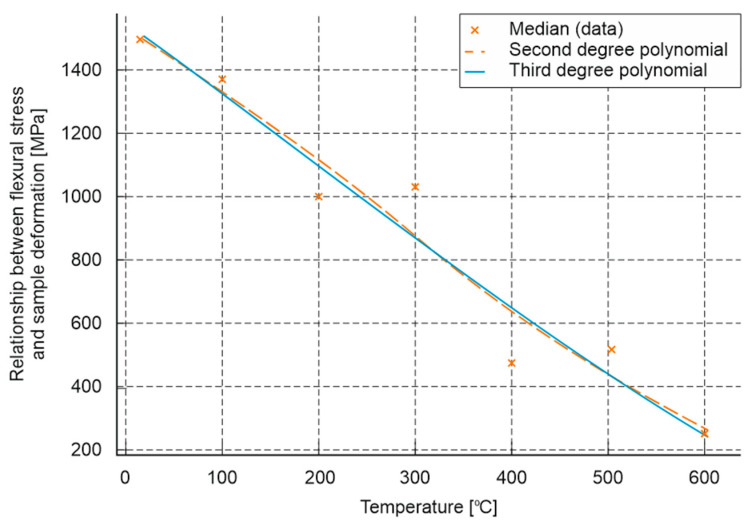
Mathematical model enabling approximate assessment of the impact of high temperature on the relationship between flexural stress and sample deformation.

Similarly to the previous studies, based on the analysis of the results, an approximate model was developed that allows the estimation of changes in the bending modulus of elasticity of cement mortar subjected to high temperatures (Figure 20). To fit the experimental data (median values at temperatures of 20, 100, 200, 300, 400, 500 and 600 °C), two mathematical models were used:

Second-degree polynomial:

(6) $$E_{b}\left(T\right)\approx 4.647\cdot 10^{-4}T^{2}-2.457T+1.5649\cdot 10^{3}$$

Third-degree polynomial:

(7) $$E_{b}\left(T\right)\approx 2.219\cdot 10^{-6}T^{3}-1.604\cdot 10^{-3}T^{2}-1.960T+1.5435\cdot 10^{3}$$

where

*E_b_(T)* denotes the flexural modulus [MPa] as a function of temperature *T* [°C].

The fitting of both models was assessed using the coefficient of determination, *R^2^*, where for the second-degree model it was *R^2^* = 0.944 and for the third-degree model *R^2^* = 0.945. In both cases, the obtained *R^2^* values are high and very close—the third-degree model provides a slightly better fit to the experimental data. The obtained relationships indicate a nonlinear character of changes in the modulus of elasticity as a function of temperature. In the analysed range, a systematic decrease in the modulus is observed with increasing temperature—the modulus value decreases from approximately 1492 MPa at 20 °C to 250 MPa at 600 °C, corresponding to a loss of about 83.2% of the initial value.

When comparing the results of the presented research with values found in the literature, it is worth referring to numerous studies that investigate the same parameters, albeit for concrete rather than mortar. For example, in his work [39], Hertz developed idealised data for concrete strength as a function of temperature and, based on an extensive synthesis of studies, identified general trends for concrete with coarse aggregate. These included an initial slight increase in strength at low temperatures (associated with the evaporation of free water and physicochemical effects in the cement), followed by a significant strength decrease. The extent and pattern of this decrease strongly depended on factors such as the type of concrete (normal- vs. high-strength), the type and proportion of aggregate, moisture content, heating rate and whether the samples were loaded during heating. Hertz provided standardised, idealised curves used in fire design, which are widely cited in reviews and engineering practice.

The main comparative observation shows that mortars exhibit the same general trend as Hertz’s curves (an increase up to ~100–200 °C, followed by a decline). However, the scale of load-bearing capacity loss observed in the present measurements was smaller than the typical reductions reported for concrete with coarse aggregate in Hertz’s studies and in review compilations. In those sources, at 500–600 °C, significantly greater reductions are often observed—depending on the type of concrete, typically in the range of 40–70% relative to room temperature values.

It is well known that mortars (cement + sand) are more microstructurally homogeneous than concrete containing coarse aggregate. The absence of thermal mismatch between the aggregate particles and the cement matrix reduces thermal stresses at the phase boundaries and, consequently, limits microcracking that would otherwise lead to a rapid loss of load-bearing capacity. Therefore, mortars may exhibit a more gradual decline in strength with increasing temperature compared to some types of concrete.

In concrete, the type and size of aggregate significantly influence the mechanical properties under investigation. For example, limestone and igneous aggregates exhibit different thermal behaviours. Coarse aggregate can lead to local concentrations of thermal stresses and spalling—that is, thermal surface flaking caused by differences in, for instance, the thermal deformation between coarse aggregate and the cement paste. In contrast, in mortar, sand introduces smaller thermal differentials. The initial increase in strength at around 100 °C is typically associated with the evaporation of free water, partial “drying out” of the structure and the crystallisation of binding phase products. Differences in water content and porosity between mortar and concrete affect the magnitude of this effect.

Similarly, smaller specimens—and therefore shallower cross-sections, as more commonly used for mortars—tend to heat more uniformly, which limits thermal gradients and associated stresses. In large concrete elements, stronger temperature gradients occur, leading to cracking and more rapid losses in load-bearing capacity.

The obtained research results also confirm the observations presented in the latest publications regarding the impact of high temperatures on the mechanical properties of cement mortars and concretes [51,52,53,54,55]. The analysis of the average bending and tensile strength pointed to an increase in these values up to 100 °C, a decrease at 200 °C, an atypical increase at 300 °C and a successive decrease at higher temperatures. These nonlinear changes correspond to the results presented by Lima et al. [51] who reported that cement-lime mortars show an initial improvement in mechanical properties under moderate heating (up to approximately 200 °C) due to further hydration and redistribution of moisture, while above 400–600 °C, a rapid degradation occurs—with a reduction in compressive strength by ca. 87% and in modulus of elasticity by over 75%. A similar trend in the low-temperature range (100–300 °C) was observed by Le et al. [52] in alkali-activated slag (AAS) mortars, where a 36.9% increase in compressive strength was recorded at 200 °C. The authors attribute this to the effect of continuous hydration and strengthening of the microstructure, which may also explain the local increase in strength observed in the analysed samples at 300 °C. From the perspective of the impact of polypropylene fibres (PP) on tensile and bending properties, the results of our own tests (a 20% increase in tensile strength for unheated samples and increases of 25% at 100 °C, 10% at 200 °C and 1% at 400 °C and 600 °C) are fully consistent with the observations for mortars and concretes with fibres presented in the literature. In the work of Ezziane et al. [53], it was demonstrated that fibres improve not only compressive and bending strength but also fracture energy and material ductility, which was attributed to the limitation of crack development and better stress distribution. In the case of our own tests, PP fibres served the same purpose, improving the tensile properties, particularly at lower temperatures, before they underwent thermal degradation (around 160–170 °C).

The mechanism of action of PP fibres, which melt and partially evaporate creating microchannels in the concrete that facilitate the dispersion of steam pressure, can be directly related to the research of Han et al. [54]. In their studies of UHPC with polyethylene fibres (PEFs), the authors achieved a porosity of 5.89% at 400 °C and eliminated the phenomenon of explosive spalling, maintaining a compressive strength of 98.3 MPa at 400 °C and 36.0 MPa at 800 °C. Although PP fibres differ from PEF in their thermal properties, the smaller decrease in bending and tensile strength observed in our own studies after exceeding 400 °C can be explained by a similar phenomenon—the formation of controlled microporosity that limits internal pressures.

The results of Kong et al. [55] for UHPC reinforced with steel-polypropylene fibres also confirm that PP fibres positively influence the mechanical properties of concrete up to around 400 °C, where a strength increase of several percentage points is observed, followed by gradual degradation at higher temperatures. The authors noted that with rapid heating, the material retains greater structural integrity, which may partly explain the small percentage differences observed in our own samples at 400 °C and 600 °C.

In summary, the obtained results confirm the typical initial improvement in mechanical properties (100–200 °C) observed in the literature [51,52], as well as local anomalies at 300 °C, which may result from water evaporation and temporary reconsolidation of the microstructure [52], and the positive effect of PP fibres on tensile and bending strength properties, consistent with the observations of authors studying UHPC with polymeric and steel fibres [53,54,55].

Despite the abundance of studies on this topic, it is clear that the present research complements the existing literature, demonstrating that in the case of mortars with polypropylene fibres, it is possible to maintain favourable mechanical properties even after exposure to 600 °C. However, the effectiveness of the fibres decreases above their melting point, yet they still significantly improve the material’s tensile and bending resistance compared to the reference mortars.

**Figure 21:**
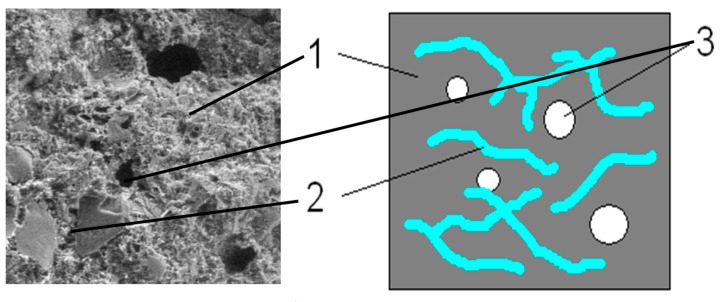
Diagram of polypropylene fibre-reinforced mortar subjected to a temperature of 100 °C. Markings: 1—cement stone; 2—PP fibres; 3—air voids formed after evaporation of water from capillaries.

In order to clarify the observed findings, SEM microscopic images of the tested composite specimens were also analysed to identify artefacts supporting the proposed assumptions. Based on the literature data, it can be stated that free water evaporates at around 100 °C, while physically bound water is removed at around 180 °C. Based on this—as confirmed by the conducted analyses—it can be assumed that water evaporation contributed to the strengthening of the cement paste structure through the densification of C-S-H gels. As a result, the cement paste itself became stronger, but the composite still exhibited structural discontinuities in the form of voids. Pores in cement composites are an inevitable and commonly occurring phenomenon. However, it may happen that they are filled with water, which makes the composite structure continuous. Due to the action of high temperatures, this water evaporates, which can potentially cause structural discontinuity in the composite. This assumption was further supported by air voids observed in the fracture surfaces of the tested specimens, as shown in Figure 21.

This hypothetical scenario suggests that tensile strength increases due to the enhanced strength of the cement paste. The voids, which under normal conditions are filled with water, do not significantly affect the tensile strength. This parameter tends to increase within the discussed temperature range. However, when analysing the same phenomenon in terms of compressive strength, the situation appears different. Water itself is compressible and can assist in transmitting compressive loads under ambient conditions. Once the water has evaporated, discontinuities emerge within the material, which can cause localised weakening of the composite structure, ultimately contributing to the observed reduction in this parameter.

**Figure 22:**
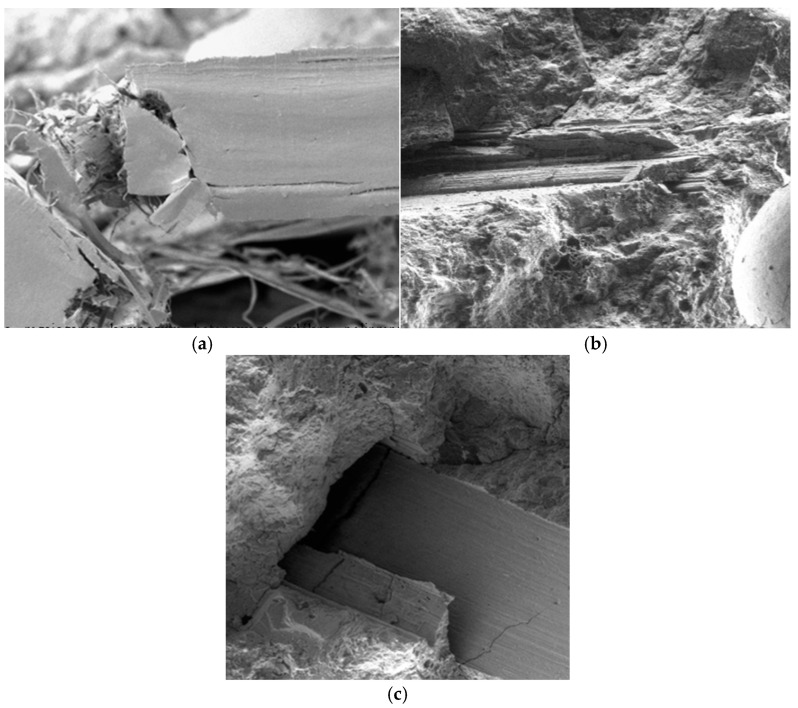
Microscopic images of PP fibre-modified composite samples at temperatures: (**a**) 20 °C; (**b**) 300 °C; (**c**) 600 °C.

The analysis of the influence of polypropylene fibres on the tested parameters across various temperatures also prompted the authors to conduct a detailed microscopic examination of their behaviour at different temperatures. SEM images of the fibres at temperatures up to 300 °C demonstrated that up to this point, the fibres retain their structure and can potentially reinforce the composite structure. Microscopic images of composite samples modified with PP fibres at temperatures of 20 °C, 300 °C and 600 °C are presented in Figure 22.

**Figure 23:**
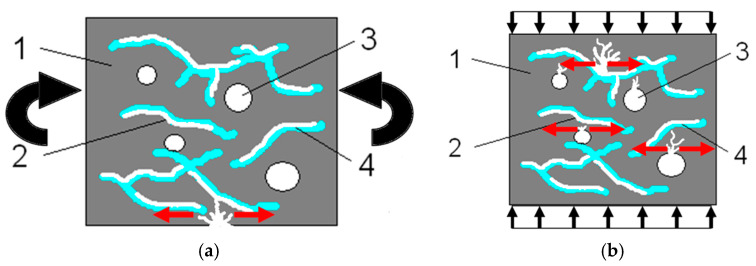
Diagram of the gradual melting of polypropylene fibres with increasing temperature: (**a**) tensile strength testing and local strengthening of the composite by the remnants of polypropylene fibres; (**b**) compressive strength testing and local weakening of the structure due to phase discontinuities caused also by the melting of fibres. Legend: 1—cement paste; 2—voids formed as a result of fibre melting—phase discontinuities causing local tensile stresses during compressive strength testing; 3—voids formed due to water evaporation; 4—parts of fibres not melted that function as dispersed reinforcement which is beneficial in terms of tensile strength testing. Red arrows indicate locally occurring tensile stresses.

Based on the SEM images, it can be stated that polypropylene fibres undergo gradual degradation over the temperature range from 20 °C to 600 °C. Up to approximately 300 °C, the PP fibres remain intact and act as dispersed reinforcement within the composite. Minor losses of fibres do not adversely affect the tensile or compressive strengths tested. Similar observations were reported by other researchers [61,62,63]. The authors of the article demonstrate that within this temperature range, the PP fibres soften, then melt and partially integrate into the cement matrix structure (penetrating the porous cement matrix), ultimately “strengthening it”. Within this temperature range, their influence is therefore beneficial. The dispersed fibres, or even their fragments, positively affect both the transfer of global tensile stresses, which improves the tensile strength of the composite, and the transfer of local tensile stresses, for example around discontinuities, which positively impacts the compressive strength of the composite. Above 300 °C, fibre loss significantly increases until their complete degradation occurs. Regarding tensile strength, even small remnants of fibres can provide beneficial reinforcement, so throughout the entire temperature range the addition of fibres has a positive effect on this property. The melting of fibres at temperatures above 300 °C causes the formation of additional phase discontinuities in the composite due to the creation of voids left by the fibres themselves. This situation is unfavourable regarding the compressive strength of the composite. The formation of these discontinuities, similarly to the air voids formed after water evaporation, can generate local tensile stresses at the boundaries of these discontinuities during compression, which leads to a decrease in this parameter. The formation of phase discontinuities after partial and complete melting of fibres is shown in Figure 23.

At a temperature of 500 °C, due to the decomposition of calcium hydroxide, which is part of the crystalline network formed during setting, chemically bound water is released as free lime (Ca(OH)_2_ → CaO + H_2_O). These processes likely led to further weakening of the structure, resulting in decreases in both compressive and tensile strength. It can be assumed that the melted polypropylene fibres could have filled microcracks in the composite occurring at the interface between the sand and the cement paste. This strengthened the cement paste structure, so tensile strength, which mainly depends on the bond between phases, was higher for the mortars with PP fibres even at elevated temperatures. Similarly, at higher temperatures, the flexural modulus of elasticity (a stiffer, less deformable composite) was higher for the composite with PP fibres. However, in the case of compressive strength testing, the voids formed after fibre melting significantly reduced the value of this parameter.

In summary, the presented microstructural observations are directly reflected in the mechanical test results. The increase in tensile strength up to around 100 °C can be associated with the densification of the C–S–H phase resulting from the evaporation of free water, which temporarily increases the cohesion of the cement matrix. In the temperature range of 100–300 °C, SEM images confirm that polypropylene fibres remain intact or are only partially melted, maintaining their ability to bridge microcracks and transfer tensile stresses locally. This phenomenon correlates with the relatively stable tensile strength and gradual decrease in compressive strength in this temperature range. Above 300 °C, the disappearance of fibres and the formation of voids observed in SEM images lead to the development of phase discontinuities, which directly explain the decrease in compressive strength due to the presence of local tensile stresses around the voids. At the same time, even residual or partially melted fibre fragments visible in the composite structure can still participate in transferring tensile loads, which justifies the more gradual decrease in tensile strength compared to compressive strength as temperature increases. The evolution of the microstructure observed in the SEM studies thus provides direct confirmation of the mechanistic causes behind the trends described in the strength tests and justifies the use of empirical models to describe these relationships.

## 4. Conclusions

The experimental work carried out on high-strength, fine-grained cement mortars modified with polypropylene fibres and subjected to simulated exposure to increasing temperatures allowed for a series of conclusions to be drawn.

Optimisation study results—the tensile strength tests performed for different types of fibres clearly indicated the positive role of dispersed reinforcement in increasing the concrete’s resistance to elevated temperatures. Fibrofor fibres demonstrated greater effectiveness across the entire tested temperature range, providing both higher initial strength and more stable behaviour during heating. Ignis fibres also positively influenced mechanical properties; however, their performance was less stable, especially around 100 °C, where a strength reduction was observed compared to the control sample.

In terms of tensile strength under flexural and splitting for both samples with and without polypropylene fibre addition, an increasing trend in this parameter was observed up to 100 °C (from 8.3 MPa to 9.6 MPa without fibres and from 10.6 MPa to 12.5 MPa with fibres), followed by an almost linear decrease down to 600 °C, reaching values of approximately 5.1 MPa (without fibres) and 5.5 MPa (with fibres). The addition of polypropylene fibres had a beneficial effect on this tested parameter throughout the entire temperature range.

As regards compressive strength testing, a practically linear decrease in strength values was observed across the entire test range (from 99.9 MPa to 73.1 MPa for composites without additives and from 110. 6 MPa to 60.3 MPa for mortars with additives), with the addition of PP fibres being beneficial up to a temperature of 300 °C and the effect of the additive being unfavourable above this temperature.

In terms of the elasticity module tested up to a temperature range of 200 °C, the analysis results proved that the composite is more compact and less deformable without fibres and that after increasing the temperature range, the composite with fibres is less deformable.

Considering the results obtained in structural elements made of mortar subjected mainly to tensile stresses, such as hollow core slabs, the use of polypropylene fibres is recommended. The effects of their use are beneficial both at room temperature and in fire conditions.

Considering the results obtained in structural elements made of mortar subjected mainly to compressive stresses, such as wall blocks, the use of polypropylene fibres in buildings exposed to fire is not recommended. Although they are beneficial at room temperature, in fire conditions they reduce the compressive strength of the composite.

For the quantitative description of the dependence of mechanical parameters on temperature, second-degree polynomial models were used, which accurately reflect the observed nonlinear trends in the decrease of strength in the range of 20–600 °C. The choice of these models was justified by their simplicity, consistency with empirical data and widespread use in the literature regarding the effect of temperature on concrete parameters.

In addition to the experimental observations, the conducted research provides a broader scientific contribution, which involves explaining the temperature-dependent mechanisms that determine the interaction between polypropylene fibres and the cement matrix. The combination of mechanical and microstructural analyses enabled a better understanding of how fibre degradation, phase discontinuities and the development of porosity collectively influence the overall strength response of high-performance mortars. The use of polypropylene fibres reduces the phenomenon of concrete spalling under fire conditions by creating microchannels that release water vapour and reduce pore pressure [70,71,72]. From a practical perspective, the results indicate, however, that the beneficial effect of polypropylene fibres for all types of structures is limited to a moderate temperature range (up to approximately 300 °C), above which the loss of fibre continuity and the formation of voids lead to a decrease in compressive strength. This finding represents a significant design limitation for fibre-reinforced cement composites used in elements exposed to fire, emphasising the need for further optimisation of fibre types and their thermal resistance in engineering applications.

The final summary of the conducted research proves that the obtained results are consistent with the general trends observed in the literature regarding the effect of high temperatures on cement mortars and concretes. They provide confirmation of the typical initial increase in strength at low temperature ranges (around 100–200 °C), resulting from additional hydration and redistribution of moisture, followed by a successive decline in mechanical properties at higher temperatures. The observed local anomalies at around 300 °C can be attributed to the temporary stiffening of the structure due to the processes of water evaporation and microscopic reconsolidation of the paste. Additionally, the observed beneficial effect of polypropylene fibres on the behaviour of the mortar under high-temperature conditions fits into the widely described mechanism of reducing the phenomenon of spalling by creating microchannels that allow for the escape of water vapour.

In this way, the own research complements the existing findings, indicating that with an appropriate fibre content of PP, it is possible to maintain relatively favourable mechanical properties of the mortar even after exposure to temperatures up to 600 °C.

## Figures and Tables

**Table 1:** Physicochemical parameters of CEM I 42.5N cement based on the technical data sheet [57].

Property	Unit	Average Result	Requirements
Start of binding	min	233	>60
End of binding	min	291	
Water demand	%	27.5	
Volume stability	mm	1.1	<10
Specific surface area	cm^2^/g	3688	
Compressive strength: after 2 days	MPa	23.9	<10
Compressive strength: after 28 days	MPa	55.9	>42.5, <62.5
Chemical analysis: SO_3_	%	2.77	<3.0
Chemical analysis: Cl	%	0.070	<0.10
Chemical analysis: Na_2_O eq.	%	0.53	<0.6

**Table 2:** Basic properties of microsilica based on the product data sheet [58,59,60].

Parameter	Unit	Value	Evaluation Method
Form	-	Fine-grained powder	Visual
Colour	-	Grey	Visual
Odour	-	Odourless	-
Density	g/cm^3^	2.05	EN 1097-6
Bulk density	g/cm^3^	1.1	EN 1097-3
Alkalinity	pH	Less than 11.5	PN-EN-ISO 10523

**Table 3:** Basic properties of the admixture based on the manufacturer’s data [63].

Feature	Description
Form	Liquid
Colour	Light brown
Density	1070 +/− 20 kg/m^3^
pH	6.5 +/− 1
Contents of Cl^−^	≤0.1%
Contents of Na_2_O	≤1.5%
Raw material base	Polycarboxylic ethers

**Table 4:** Characteristics of Ignis and Fibrofor High Grade fibres used in the tests [63].

Property	Fibre Name	
	Ignis	Fibrofor High Grade 190
Colour	Transparent	Beige
Characteristic	Monofilament	Bound, fibrillated
Length, [mm]	12	19
Film thickness, [μm]	18	80
Density, [g/cm^3^]	0.91	0.91
Tensile strength, [N/mm^2^]	min 28 cN tex^−1 a^	~400
Softening temperature, [°C]	~165	~150

^a^ cN tex^−1^—tensile strength unit for fibre, where tex is a unit for the linear mass density of fibres.

**Table 5:** Cement mortar compositions.

Components	Abbreviation		
	0F	1.8F	1.8I
Cement CEM I 42.5 R, [kg/m^3^]	846	846	846
Silica, [kg/m^3^]	84.6	84.6	84.6
Sand, [kg/m^3^]	1249	1249	1249
Optima 185 plasticiser, [%]cement mass	2	2	2
Water, [dm^3^]	215	215	215
Polypropylene fibres, [kg/m^3^]	0	1.8	1.8

**Table 6:** Results of tests on the relative change in flexural modulus as a function of temperature for mortar with and without the addition of polypropylene fibres “F” in an amount of 1.8 kg/m^3^ (compared to the flexural modulus at 20 °C).

Mortar Type	Average Flexural Modulus of Elasticity in Flexural Δσ/Δε, [MPa]							
		20 °C	100 °C	200 °C	300 °C	400 °C	500 °C	600 °C
Z0F	E_av_ [MPa]	1489	1415	904	809	633	577	272
	E_av_ T/E_av_ 20 °C [%]	100	95.1	60.8	54.3	42.5	38.8	18.3
	Decrease [%]	0	4.9	39.2	45.7	57.5	61.2	81.7
Z1,8F with 1.8 kg/m^3^ F	E_av_ [MPa]	1237	1113	1006	882	771	606	288
	E_av_ T/E_av_ 20 °C [%]	100	90.0	81.4	71.3	62.3	49.0	23.3
	Decrease [%]	0	10.0	18.6	28.7	37.7	51.0	76.7

Note: Eśr20 °C—flexural modulus of elasticity of the tested mortar at 20 °C.

## Data Availability

The data have been archived in Faculty of Safety Engineering and Civil Protection, Fire University, 52/54 Słowackiego St., 01-629 Warsaw, Poland. Further inquiries can be directed to the corresponding author.
